# After the Nozzle: Post-Printing Maturation, Failure Modes, and Use-Point Assessment of Cell-Laden Extrusion-Bioprinted Hydrogel Constructs

**DOI:** 10.3390/gels12080742

**Published:** 2026-08-19

**Authors:** Yifan Li, Xiuyu Wen, Linken Li, Li Li, Jianghong He

**Affiliations:** Key Laboratory of Neuroregeneration of Jiangsu and Ministry of Education, Co-Innovation Center of Neuroregeneration, NMPA Key Laboratory for Research and Evaluation of Tissue Engineering Technology Products, Nantong University, Nantong 226001, China; liyifan198@hotmail.com (Y.L.);

**Keywords:** extrusion bioprinting, cell-laden hydrogel bioink, post-printing maturation, use-point assessment, construct failure, longitudinal characterization, tissue engineering

## Abstract

Cell-laden hydrogel bioinks for extrusion bioprinting are commonly evaluated by precursor rheology, extrusion behavior, and immediate shape fidelity, yet these measures do not establish whether printed constructs remain stable and functional during culture or tissue maturation. This review examines the post-printing evolution of cell-laden extrusion-printed hydrogel constructs, prioritizing direct evidence from cell-laden prints and using acellular prints and bulk hydrogels only to clarify mechanisms. Maturation is organized into immediate stabilization, network evolution and environmental equilibration, and long-term remodeling. Crosslinking, hydration, ion exchange, stress relaxation, degradation, cellular contraction, and matrix deposition may support maturation or cause structural, mechanical, interfacial, transport, and biofunctional failure. We propose a conceptual, research-oriented use-point assessment framework that compares each construct with relevant reference states and application-specific requirements after stabilization, during culture, and before intended use. The framework links the earliest observed critical deviation to relevant measurements, targeted redesign, and reassessment under the same conditions. A cartilage construct illustrates sequential evaluation of geometry, wet-state mechanics, cell distribution, and matrix formation. Current evidence is limited by inconsistent assessment times, incomplete reporting, and few integrated longitudinal studies. Future work should standardize maturation histories, model construct trajectories, and prospectively validate product-specific criteria. Evaluation should focus on the complete cell-laden extrusion-printed construct at its intended use point rather than on the freshly printed filament.

## 1. Introduction

Cell-laden hydrogel bioinks are widely used in extrusion-based bioprinting because they combine hydrated cell support with adjustable rheological and biochemical properties. Current assessment nevertheless remains strongly centered on precursor rheology, extrusion behavior, and immediate shape fidelity. Viscosity, shear-thinning behavior, yield stress, recovery, filament diameter, spreading, and collapse resistance are important because they describe material delivery and early structural retention [1,2]. These printability-related outcomes are governed not only by precursor rheology but also by formulation and crosslinking conditions, which influence filament formation, immediate shape retention, and short-term cell viability, as demonstrated in gelatin methacryloyl (GelMA) and photocrosslinkable collagen bioinks [3,4]. These measurements establish whether a bioink can be printed, but they do not determine whether the resulting cell-laden construct will remain stable and functional during later use.

This limitation arises because, after deposition, the construct continues to change through crosslinking, hydration, ion exchange, degradation, stress relaxation, and cell-mediated remodeling. These processes can improve filament integration and tissue formation, but they can also cause swelling, pore closure, softening, contraction, or loss of structural continuity. Bulk-hydrogel studies show that stress relaxation can regulate stem-cell behavior independently of initial elastic properties [5]. Direct longitudinal assessment of cell-laden three-dimensional (3D) bioprinted hydrogel scaffolds further showed that whole-construct mechanical properties evolved over 7 days as swelling, material loss, and cell proliferation altered the construct response [6]. Together, these findings explain why immediate printability cannot predict the later structural, mechanical, or biological state of a construct, although direct longitudinal testing remains necessary for the target cell-laden system.

Delayed failure is particularly evident in time-dependent networks. In a high-cell-density gelatin-hyaluronic acid (HA) system crosslinked with genipin, one formulation fully dissolved within 48 h in phosphate-buffered saline at 37 °C, whereas the other formulations showed partial disintegration over the same interval [7]. Rapid photocrosslinking can provide immediate support, although the resulting network depends on light dose, photoinitiator concentration, functionalization, construct thickness, and oxygen availability [8,9]. Dynamic covalent networks may improve recovery and filament integration while continuing to reorganize after deposition. GelMA and hydrazone-linked HA double networks illustrate how reversible and permanent bonds can jointly support extrusion, integration, and cell compatibility [10]. These examples show that successful deposition marks only the initial state and does not demonstrate sustained performance.

Early-stage assessment has recently advanced through quantitative artifact analysis, optical measurement, in-process imaging, and predictive modeling [11,12,13,14]. These approaches improve process control and defect detection. However, post-printing evidence is still commonly reported as separate measurements of swelling, degradation, mechanics, viability, transport, or tissue-specific activity. Many studies evaluate one formulation, exposure condition, or endpoint and do not connect the timing of change with construct-level failure and the state required before subsequent handling, culture, perfusion, loading, or implantation. Consequently, favorable printability or an isolated post-printing result may be interpreted without considering the complete maturation trajectory.

**Figure 1:**
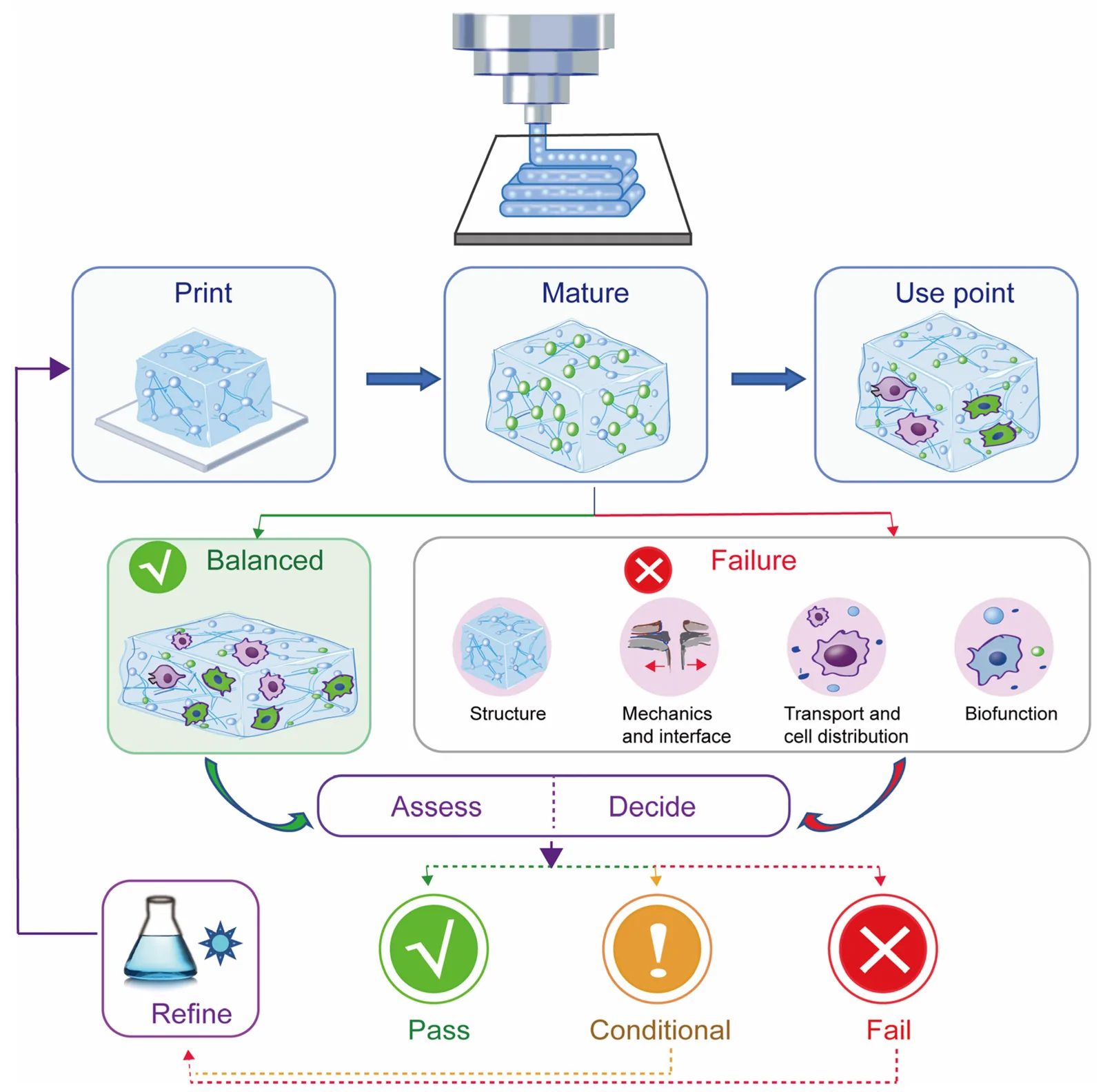
Conceptual overview of post-printing maturation, failure-sensitive assessment, and iterative bioink refinement at a defined use point. Assessment modules are application-specific and may be applied in parallel, reordered, or omitted. Pass, Conditional, and Fail are defined in Section Operational Decision Rules. Redesign in one domain requires reassessment of other affected domains.

The literature on hydrogel bioinks is frequently organized by polymer source, crosslinking mechanism, rheological behavior, or initial printing fidelity. This organization supports material selection but does not fully explain how network evolution after printing progresses to construct-level failure and functional loss. Assessments performed at unrelated time points or under different exposure histories are also difficult to compare. A longitudinal framework must distinguish the freshly printed state, immediate stabilization, environmental equilibration, and longer-term remodeling while relating each state to the requirements of the next intended use. Here, a use point refers to the application-specific time and condition at which the construct is evaluated against its required performance. This perspective shifts assessment from immediate printability to the state of the construct when it must be handled, cultured, loaded, or used. The overall framework is summarized in Figure 1.

### 1.1. Scope and Boundaries

This review focuses on hydrogel constructs produced by extrusion bioprinting in which living cells are incorporated before or during deposition. Conventional free-standing extrusion, embedded extrusion, coaxial extrusion, multimaterial extrusion, sacrificial strategies that create perfusable channels, and extrusion of microtissue-containing hydrogel formulations are included when the resulting cell-laden hydrogel construct undergoes post-printing maturation. Inkjet, laser-assisted, and vat-photopolymerization methods are outside the primary scope and are considered only when they provide mechanistic context that is directly relevant to post-printing hydrogel behavior. Accordingly, conclusions are intended for cell-laden extrusion-printed hydrogel constructs and should not be generalized to all bioprinting modalities.

### 1.2. Positioning and Novelty of the Use-Point Framework

Baseline comparison, predefined performance criteria, longitudinal characterization, failure identification, and iterative redesign are established concepts in quality by design, critical quality attribute assessment, process–structure–property analysis, tissue-engineering evaluation, and product development. The present framework does not claim these elements as individually novel or validated. Its contribution is their construct-centered integration after extrusion, including explicit separation of printing-origin conditions from post-printing change using multiple reference states, organization of overlapping maturation processes by dominant time regime, mapping of observed deviations to structural, mechanical and interfacial, transport and cell-distribution, and biofunctional domains, use-point-specific decision rules, and identification of the earliest observed critical deviation within the available temporal resolution to guide targeted redesign. The framework is therefore a research-oriented synthesis for organizing post-printing evidence, not a regulatory qualification method or a universally validated specification system. Recent extrusion-bioprinting studies have also integrated real-time quality monitoring with automated error correction, showing that process monitoring and feedback control are established components of quality-oriented bioprinting rather than unique elements of the present framework [15].

### 1.3. Literature Identification, Study Selection, and Evidence-Directness Classification

Literature identification was structured around PubMed, Web of Science Core Collection, and Scopus, with backward citation checking of included research articles. The search covered publications from 1 January 2016 through the final search date of 9 August 2026, and only English-language records were considered. The search combined three concept blocks covering the printing modality, the cell-laden hydrogel or bioink, and post-printing maturation or outcome terms. Complete database-specific search strings are provided in Table A3.

Eligible primary research articles examined extrusion-printed cell-laden hydrogel constructs or, when direct evidence was unavailable, acellular-printed constructs or bulk hydrogels that addressed a specific post-printing mechanism. Reviews, editorials, conference abstracts, studies without hydrogel relevance, and nonextrusion studies without direct mechanistic relevance were excluded. Regulatory, standardization, and policy documents were retained separately for Section 5.3 and were not included in the research-study count. Duplicate records were checked in EndNote using DOI, title, first author, and publication year followed by manual verification. Records were screened by title and abstract and then by full text.

The database searches identified 1986 records before deduplication. After removal of 917 duplicate records, 1069 unique records underwent title or abstract screening. Of these, 132 articles were assessed in full text, and 69 were excluded after full-text assessment because they did not meet the eligibility criteria. The final primary-study evidence set comprised 63 primary research studies, as detailed in Table A1.

Each primary research study was assigned one primary evidence-directness class according to the evidence most directly used in this review. E1 denotes longitudinal cell-laden extrusion-printed constructs with at least two post-printing time points capable of establishing a direction of change, E2 denotes endpoint, immediate, or otherwise non-trajectory cell-laden extrusion-printed constructs, E3 denotes acellular extrusion-printed constructs or acellular constructs that were seeded after printing, E4 denotes bulk cell-laden hydrogels, and E5 denotes acellular bulk hydrogels. Studies that also contained a computational or machine-learning component retained the relevant E1–E5 class and were additionally marked with a C modifier; C alone denotes a purely computational study. These classes describe applicability and directness to the review question rather than methodological quality, risk of bias, or certainty of evidence. E1 provides the most direct evidence for maturation trajectories, whereas E3–E5 are used for architectural or material mechanisms and are not treated as quantitative surrogates for cell-laden printed constructs. Study-level extraction included bioink composition, crosslinking, cell type and density, printing mode, architecture, culture or exposure conditions, assessment time points, structural, mechanical, transport, and biological measurements, observed post-printing behavior, longitudinal design, repeated-measure status, replicate reporting, and spatial versus whole-construct assessment. Table A1 provides the extracted evidence. Items not available in the reviewed report are marked NR.

## 2. Temporal Stages of Post-Printing Maturation

**Figure 2:**
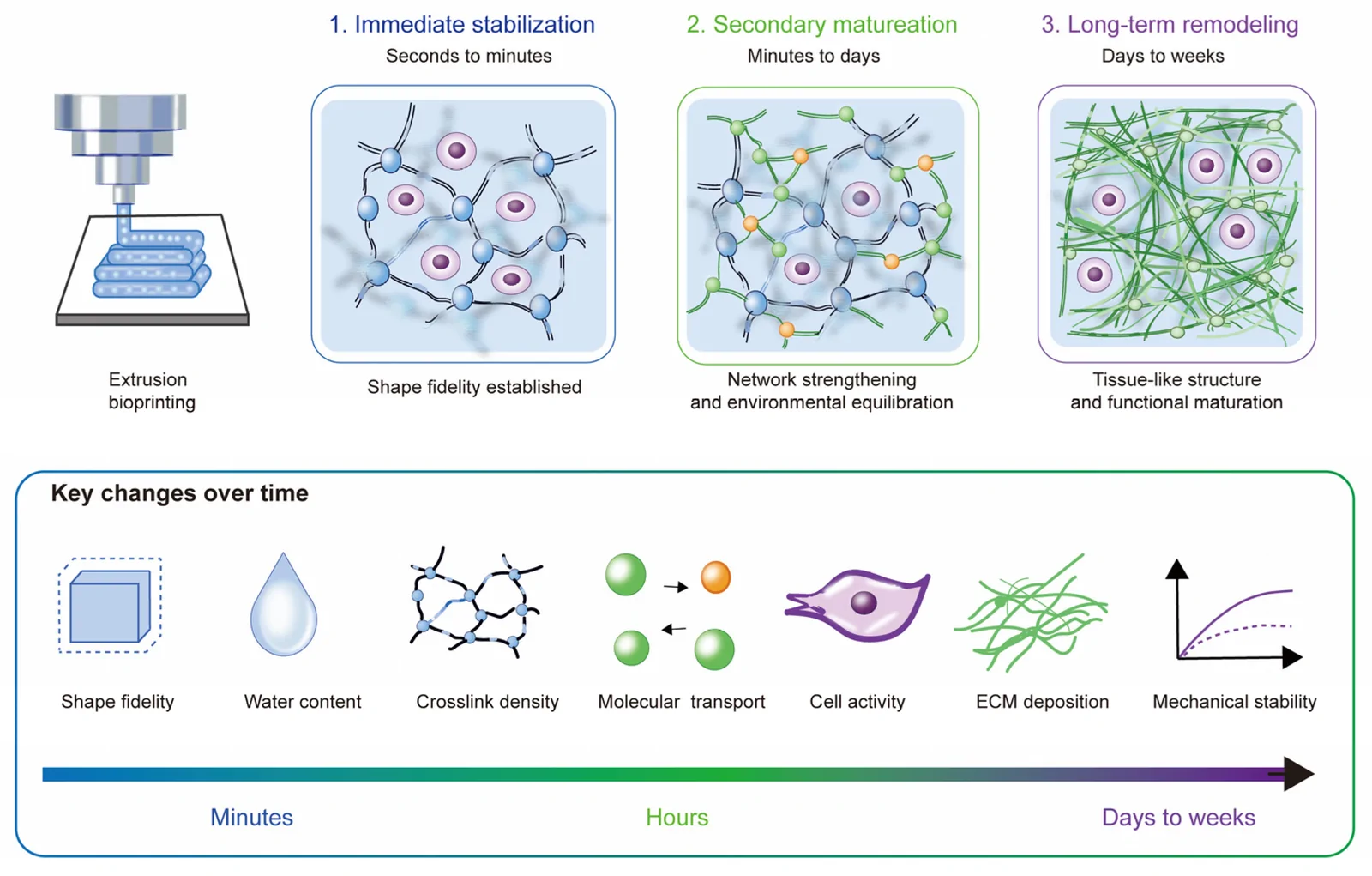
Temporal stages of post-printing maturation and representative structural, transport, cellular, and mechanical changes in cell-laden hydrogel constructs. The time labels and mechanical trajectories represent conceptual process regimes rather than universal measured trends. Processes may overlap and vary with material chemistry, construct dimensions, and culture conditions.

In this review, post-printing maturation refers to the material and biological changes that occur in a cell-laden extrusion-printed hydrogel construct after deposition. This section describes how the construct changes over time without assigning success or failure. Immediate stabilization usually dominates within seconds to minutes, secondary network formation and environmental equilibration over minutes to days, and long-term remodeling over days to weeks or longer. These ranges overlap and indicate dominant processes, not fixed limits. The temporal sequence and associated changes, including extracellular matrix (ECM) deposition, are summarized in Figure 2.

These intervals are heuristic process regimes rather than universal boundaries. Dominant effects may be immediate, including flow recovery and rapid thermal, ionic, or photochemical stabilization. Intermediate effects include continued crosslinking, ion exchange, hydration, and soluble-fraction loss, whereas delayed effects include degradation, cell-driven contraction, and extracellular matrix deposition. The same process may span more than one regime, and its rate depends on material chemistry, construct dimensions, exposure history, and cell activity. Representative overlapping time scales are summarized in Table A2.

### 2.1. Immediate Post-Deposition Stabilization

The first stage begins as a cell-laden bioink exits the nozzle and the deposited filament recovers from the deformation imposed during extrusion. Shear-thinning facilitates flow through the nozzle, whereas rapid structural recovery, immediate external stabilization, or a combination of both is required after deposition to restrict spreading, preserve filament continuity, and support subsequent layers. Intrinsic recovery may involve chain re-entanglement, reformation of hydrogen bonds, hydrophobic association, supramolecular assembly, or restoration of other reversible physical interactions.

Thermal gelation can provide additional temporary support. Gelatin-containing systems illustrate this function because cooling promotes physical association and improves deposition, whereas the resulting network weakens at physiological temperature unless it is subsequently reinforced. Direct cell-laden extrusion studies have shown that optimized reversible physical crosslinking can improve the deposition of chondrocyte-laden GelMA before permanent ultraviolet stabilization [16]. Complementary acellular evidence shows that methylcellulose can provide temporary rheological and deposition support, whereas photocrosslinked GelMA maintains the printed structure in biological medium [17].

Beyond intrinsic recovery and thermal support, rapid ionic or photochemical crosslinking may stabilize the filament during deposition or immediately afterward. Calcium-mediated alginate crosslinking is a common example of rapid ionic fixation, although the resulting ionic state may continue to evolve after transfer to culture medium. Initial light exposure can rapidly stabilize methacrylated polymers, but the extent and uniformity of conversion depend on light dose, photoinitiator concentration, oxygen inhibition, construct thickness, and optical attenuation. Bulk GelMA experiments further indicate that photoinitiator chemistry and oxygen-related effects influence reaction kinetics and network formation, while the same study used a 6 s light exposure before the first real-time modulus measurement, illustrating that photochemical network formation can begin on a seconds timescale [9]. However, these mechanistic findings require direct confirmation in cell-laden printed geometries.

A dual-stage strategy can assign extrusion support to pre-gelation and rapid shape fixation to light exposure during or immediately after deposition. In dual-gelation hyaluronan bioinks, enzymatic pre-gelation produces a soft gel suitable for extrusion, whereas visible-light crosslinking reinforces the deposited filament [18]. These mechanisms therefore serve different temporal functions. Gelatin-based thermoreversible association provides rapid but temporary support, ionic crosslinking provides rapid fixation that remains sensitive to subsequent ion exchange, and photocrosslinking offers more persistent stabilization but may produce spatially nonuniform conversion. The endpoint of this initial stage is therefore a construct that is sufficiently stabilized for subsequent handling or culture, rather than a mature or uniformly equilibrated network.

### 2.2. Secondary Network Formation and Environmental Equilibration

After initial stabilization, two processes continue to change the construct. Secondary network formation increases or reorganizes network connectivity, whereas environmental equilibration changes hydration and composition through exchange with the surrounding medium. These processes occur concurrently but have different effects on the post-printing state.

Secondary network formation may involve delayed enzymatic or covalent crosslinking. In photocrosslinked GelMA constructs, post-printing microbial transglutaminase treatment increased stiffness, reduced swelling, and improved geometric stability [19]. Dynamic covalent bonds may also continue to exchange after printing. In cell-laden single- and double-network HA hydrogels, reversible bonds supported extrusion and recovery, whereas the permanent network provided greater mechanical stability after deposition [20]. Small-molecule modulators can further adjust bond-exchange kinetics and network formation [21].

Environmental equilibration begins as water, mobile ions, and soluble network fractions move between the construct and the culture medium. After initial calcium-mediated stabilization, acellular alginate–gelatin constructs showed treatment-dependent swelling and degradation [22]. Cell-laden alginate bioinks also underwent continued cation exchange, with associated changes in mechanics, degradation, cell response, and matrix accumulation [23]. The post-printing state therefore depends on the balance between continued network formation and material exchange with the surrounding environment.

### 2.3. Long-Term Construct Remodeling

Long-term remodeling follows early stabilization and environmental equilibration, although these stages may overlap. During this period, stress relaxation, degradation, cellular forces, and ECM deposition progressively change the structure and mechanical support of the construct. For degradable tissue-forming constructs, successful remodeling requires loss of hydrogel support to be balanced by sufficient tissue formation.

Stress relaxation allows the network to dissipate cell-generated stress through water movement, bond exchange, and chain rearrangement. Bulk hydrogel studies show that faster relaxation can promote cell spreading, matrix remodeling, and cell fate regulation independently of the initial elastic modulus [5,24]. These studies provide relevant cell–matrix mechanisms but do not directly describe printed constructs. Extrusion-induced shear can orient polymeric or dispersed network components, while printing and post-processing can generate spatial differences in crosslink density. Stress-relaxation findings from homogeneous bulk gels should therefore be treated as mechanistic guidance rather than directly transferable quantitative rules for printed constructs [25,26].

Degradation and cellular forces produce more visible construct-level changes. Network loss can create space for cell migration and matrix deposition, but premature degradation may reduce support before sufficient tissue has formed. Cell traction and proteolytic activity may also cause contraction. In cell-laden collagen constructs produced by embedded extrusion bioprinting, physically assembled networks underwent substantial cell-induced contraction during culture, whereas bioorthogonal covalent crosslinking improved long-term shape retention [27].

Direct cartilage studies show that degradation can support rather than impair maturation when new tissue replaces the hydrogel. Cartilage microtissues remained viable and fused within rapidly degrading oxidized-alginate bioinks [28]. In chondrocyte-laden gelatin–alginate constructs, optimization of bioink composition and stiffness preserved long-term morphological fidelity and increased type II collagen expression, indicating that structural maintenance can coexist with cartilage matrix formation during culture [29]. In bone-oriented constructs, hydroxyapatite reduced swelling and degradation while promoting osteogenic differentiation and mineralization, showing that a persistent reinforcing phase can shift this balance [30].

Long-term remodeling should therefore be judged by whether hydrogel loss, cellular remodeling, and new tissue formation together preserve the required construct structure and function.

## 3. Failure Domains Arising from Unbalanced Post-Printing Maturation

The changes described in Section 2 become failures only when they violate a predefined construct requirement at a specified time and condition. Because one maturation process can affect several outcomes, classification by mechanism alone creates overlap. This section defines four outcome domains: structural and dimensional failure, mechanical and interfacial failure, transport and cell-distribution failure, and biofunctional failure. The initiating process is recorded separately. At each use point, the primary domain is assigned according to the earliest observed construct-level criterion that exceeds its predefined limit, not an assumed causal origin. Secondary consequences are reported separately. If two criteria are crossed within the same assessment interval, or if temporal resolution or measurement uncertainty prevents ordering, they should be reported as co-primary failures.

Swelling, degradation, ion exchange, bond exchange, and cell-mediated contraction are maturation processes rather than failure domains. They enter the classification only when they cause an unacceptable response. Contraction is assigned to the structural domain when dimensional tolerance is violated first and to the biofunctional domain only when biological performance fails first. When cell-driven contraction causes structural and biofunctional criteria to be crossed within the same assessment interval and their sequence cannot be resolved, the event should be classified as a co-primary failure. In cartilage constructs, this may involve dimensional contraction together with inadequate matrix maturation, whereas in bone constructs, deformation may coincide with impaired osteogenic maturation or mineralization. Delamination is assigned to the interfacial subdomain when boundary continuity is lost first. A cell-density gradient present immediately after deposition is recorded as a printing-origin condition. It becomes a post-printing failure only when it remains above, worsens beyond, or newly exceeds the use-point criterion. Here and throughout, “first” refers to the earliest observed criterion within the assessment schedule rather than proof of the underlying causal sequence.

### 3.1. Structural and Dimensional Failure

Structural and dimensional change becomes failure when the hydrated construct no longer maintains the required dimensions, shape, layer continuity, pores, or channels. Manifestations include dissolution, progressive filament fusion, pore closure, warping, shrinkage, and dimensional drift. Geometry loss during hydration or culture is assigned to this domain, whereas deformation caused only by a defined mechanical challenge is assigned to bulk mechanical failure.

Swelling, degradation, ion exchange, and cell-mediated contraction can initiate these changes. In cell-laden GelMA–alginate constructs, polymer composition and combined ionic and covalent crosslinking regulated swelling and degradation [31]. Post-printing ionic treatment may alter swelling and structural lifetime [22]. Cell-generated forces caused substantial contraction in bioprinted hydrogels, whereas shrink-resistant collagen–hyaluronan networks improved dimensional retention [32,33].

Mitigation should address the dominant cause by adjusting network composition, crosslinking conditions, ionic treatment, or resistance to cell-mediated contraction. The revised construct should be assessed against both the reference geometry and the geometric requirement specified for the use point.

### 3.2. Mechanical and Interfacial Failure

Bulk and interfacial failures are grouped because both impair construct-level load transfer. They are assessed separately because acceptable bulk behavior can coexist with local interface failure.

#### 3.2.1. Bulk Mechanical Failure

Bulk mechanical change becomes failure when the hydrated construct no longer provides the load response required for handling, conditioning, or function. Relevant manifestations include progressive softening, excessive creep, incomplete recovery, residual deformation, fatigue damage, buckling, and collapse. A change in modulus or relaxation is not a failure unless the predefined mechanical requirement is violated.

Available studies mainly provide evidence for mechanical design strategies rather than longitudinal failure during maturation. Cell-laden self-healing hydrogels have shown improved toughness and fatigue resistance, while elastin-containing double-network bioinks provide a mechanically reinforced platform for extrusion bioprinting [34,35]. Human bone allograft particles also increased stiffness and reduced swelling in cell-laden composite bioinks [36]. For quantitative context, uniaxial tensile testing of a cell-laden collagen-only biopatch gave an elastic modulus of 12.1 ± 1.3 kPa, whereas an acellular direct ink writing hydrogel study reported tensile Young’s moduli of 0.193–1.072 MPa [37,38]. Because these values were obtained from different materials and specimen/test configurations, they provide only order-of-magnitude context and are not directly comparable or usable as universal acceptance thresholds.

Mechanical measurements should be reported with the test mode and data-reduction definition. Compressive modulus should specify the strain interval and whether a secant, tangent, or other definition is used. Oscillatory testing should report storage and loss moduli with frequency and strain amplitude. Stress-relaxation testing should report imposed strain and a defined time constant, half-time, or normalized relaxation. Creep testing should report applied stress and compliance. Cyclic testing should report loading amplitude, frequency, cycle number, preconditioning, recovery, and failure criterion. Toughness should be derived from a stated stress–strain protocol. Interfacial strength is a local interface metric and should not be inferred from bulk modulus.

Measurements should be performed in the relevant hydrated medium and temperature and should report sample dimensions, geometry, boundary conditions, strain or loading rate, loading history, instrument settings, normalization method, and equilibration time. Destructive tests require companion specimens matched for batch and maturation history, whereas nondestructive methods can support repeated measurements of the same construct but require validation against the target mechanical property [6,39].

Mitigation should target the inadequate response, such as stiffness, recovery, creep resistance, or fatigue resistance. The revised construct should be reassessed after the same maturation and loading history, together with the relevant cell and matrix responses.

#### 3.2.2. Interlayer or Multimaterial Interface Failure

Interfacial change becomes failure when a critical interlayer or multimaterial boundary loses continuity, separates, cracks, or no longer transfers load adequately. Delamination is assigned to this subdomain when interface continuity or load transfer is the first requirement violated.

Interface performance is strongly influenced by printing and stabilization, although failure may become evident only after swelling, degradation, or loading. In acellular extrusion-printed dual-network hydrogels, simultaneous post-processing curing formed a more continuous network and improved tensile performance compared with layer-by-layer curing [40]. Chemical coupling strengthened printed hydrogel–elastomer interfaces [41]. Mechanically interlocked hydrogel–polylactide (PLA) interfaces also improved interfacial strength and toughness, although failure remained concentrated in the softer hydrogel phase [42]. These studies provide interface-design evidence but do not directly describe longitudinal interface failure in cell-laden constructs.

Mitigation may involve adjustment of the curing sequence, introduction of compatible interfacial chemistry, or mechanical interlocking. Interface performance should be measured locally after the relevant maturation and loading history because bulk mechanical measurements cannot determine local continuity or load transfer.

Four interface classes should be distinguished: interlayer boundaries within one material, boundaries between different hydrogel formulations, hydrogel-support or hydrogel-polymer boundaries, and tissue-construct boundaries formed during maturation or implantation. Their failure mechanisms are not interchangeable. Crosslinking sequence, time between layer depositions, surface dehydration, swelling mismatch, stiffness mismatch, degradation mismatch, and cell-driven remodeling may alter local stress transfer. Depending on geometry, quantitative assessment can use peel, lap-shear, tensile-adhesion, fracture-energy, or local imaging methods, with test direction and wet-state history reported. A cell-laden multimaterial bionic construct maintained a sensor–tissue interface throughout 10 days of tissue development while preserving high cell viability and differentiation capacity, providing a direct example of interface stability during maturation [43]. Nevertheless, direct longitudinal cell-laden interface evidence remains sparse, so current interface recommendations remain largely mechanistic and should not be treated as validated quantitative thresholds [40,41,42].

### 3.3. Transport and Cell Distribution Failure

Transport and cell distribution become failures when the construct no longer maintains the required exchange capacity or regional cell status. Relevant signs include channel narrowing or occlusion, increased flow resistance, leakage, inadequate oxygen or nutrient delivery, regional cell loss, persistent cell-density gradients, and nonuniform ECM deposition.

Post-printing transport depends on whether the original pores and channels remain functional during swelling, degradation, cell growth, and matrix deposition. A perfused 3D hydrogel vascular model showed that channel permeability and endothelial barrier function require direct measurement [44]. Longitudinal monitoring of centimeter-scale, fibroblast-laden bioprinted tissues further linked changes in oxygen, metabolism, construct morphology, and internal perfusion during culture [45]. Whole-construct averages may therefore conceal regional transport failure.

Mitigation should address the cause of transport loss. Channel geometry, construct thickness, crosslinking, degradation, or perfusion conditions may be adjusted according to whether the deviation results from channel closure, excessive diffusion distance, or inadequate flow. The revised construct should be reassessed using regional measurements of permeability, oxygenation, cell viability, and matrix distribution.

Transport should be quantified separately for diffusion-dominated and perfused constructs. Candidate metrics include effective diffusivity (m^2^ s^−1^), permeability (m^2^), pressure drop (Pa), hydraulic resistance, flow rate, leakage, oxygen partial pressure or concentration, nutrient gradients, and accumulation of metabolites or waste products. Interpretation requires construct thickness, cell density and oxygen-consumption demand, pore interconnectivity, channel geometry, perfusion rate, and extracellular matrix accumulation. Spatial oxygen sensors, tracer imaging, perfusion imaging, metabolic measurements, and computationally supported concentration profiles can reveal regional limitations that endpoint viability cannot resolve [44,45,46]. In cell-laden oxygenating colloidal constructs, oxygen release was measured directly together with printability, mechanical properties, and cell viability, illustrating the value of treating oxygen availability as a measurable construct variable [47]. Preserved channel diameter alone is therefore insufficient evidence of transport adequacy.

### 3.4. Biofunctional Failure

Biofunctional change becomes failure when the intended tissue-specific activity is not achieved at the specified use point despite acceptable geometry, mechanical integrity, transport, and general viability. Relevant outcomes include inadequate matrix formation, undesired differentiation, loss of phenotype or zonal identity, failed tissue fusion, and ineffective bioactive delivery.

Degradation should be interpreted together with tissue formation. Complementary alginate-based studies illustrate this coupling: one used rapid network loss to enable cartilage microtissue fusion and glycosaminoglycan (GAG)-rich tissue formation, whereas another tuned degradation to support cartilage tissue development [28,48]. Bioactive delivery should likewise be judged by both release behavior and the resulting tissue response. Controlled delivery of transforming growth factor beta 1 (TGF-β1) and fibroblast growth factor 18 (FGF-18) from a cell-containing bioprinted scaffold promoted chondrogenesis and cartilage regeneration [49].

Mitigation should target the failed function. This may require adjustment of degradability, cell or microtissue density, biochemical induction, or bioactive delivery. The revised construct should be reassessed using the same tissue-specific endpoint.

Live/dead imaging should be treated as one component rather than a stand-alone measure of biological function. It may not detect reduced metabolic activity, apoptosis, senescence, inflammatory activation, altered potency, or phenotypic drift. In cell-laden printed alginate–gelatin constructs, live/dead staining and ATP measurements gave non-equivalent but complementary information, supporting combined assessment rather than single-assay interpretation [50]. Application-specific panels should combine viability and cell number with molecular, biochemical, histological, and functional endpoints and normalize matrix production, when appropriate, to DNA, cell number, or another predefined denominator. A 28-day stem-cell-laden bioprinting study also combined metabolic activity with extracellular matrix and lineage-related protein measurements, providing a direct example of multi-endpoint biological assessment during maturation [51].

### 3.5. Chemical, Biochemical, and Microbiological Safety as a Cross-Cutting Gate

Post-printing chemical and microbiological hazards are treated as a cross-cutting gate rather than a fifth performance domain because they can affect all four domains and can independently prevent progression. Relevant exposure history includes photoinitiator and crosslinker concentration, light and thermal dose, conversion of reactive groups, residual crosslinkers or leachables, degradation products, ionic composition, pH, osmolality, and retained bioactive-factor potency. Higher photoinitiator loading can increase network stiffness while increasing reactive oxygen species and reducing viability in cell-laden GelMA systems, illustrating why mechanical benefit cannot be interpreted independently of chemical exposure [52].

Microbiological controls should be defined according to the intended use and may include sterility or bioburden, mycoplasma, and endotoxin. Endotoxin contamination can alter inflammatory cell behavior and apparent therapeutic response in 3D-printed cell models [53]. General viability cannot substitute for these measurements. A non-negotiable safety criterion that is exceeded should be classified as a gatekeeping failure. Progression resumes only after the exposure, washing or equilibration, aseptic processing, storage, or bioactive loading step has been revised and the complete pre-use pathway has been repeated. Direct longitudinal evidence for cell-laden extrusion-printed products remains limited, so product-specific analytical and microbiological validation is required.

### 3.6. Coupled and Time-Dependent Failure Trajectories

The four failure domains may develop in sequence. The primary domain is assigned according to the earliest observed construct-level requirement that is violated within the available assessment schedule. If the order cannot be determined, the affected domains are reported as co-primary failures. For example, swelling may first be observed as a geometric deviation and later coincide with mechanical, transport, or biological failure. The same domain names are used as evaluation modules in Section 4, where they are applied at sequential and application-specific use points.

Temporal assignment should be based on the interval bounded by the last observation that met a criterion and the first observation that did not. The apparent earliest failure can therefore shift with sampling frequency, assay sensitivity, missing measurements, or measurement uncertainty. The assessment schedule, detection limits, and uncertainty should be reported. When affected intervals overlap or ordering cannot be resolved, co-primary classification is used and causal attribution is avoided. The time and location of the earliest observed critical deviation may guide hypothesis generation for the initiating process, but redesign should target only a mechanism supported by the available evidence. The revised construct should then be reassessed at the same use point under the same conditions. Table 1 summarizes principal responses and trade-offs.

**Table 1:** Failure signals, targeted responses, and principal trade-offs across post-printing failure domains and subdomains.

Failure Domain or Subdomain	Main Failure Signal	Targeted Response	Principal Trade-Off	Representative Evidence
Structural and dimensional	Dimensions, pores, channels, or shape no longer meet the specified requirement	Adjust network composition, crosslinking, ionic exposure, or resistance to cell-mediated contraction	Greater stabilization may restrict transport and tissue remodeling	[22,31,33] (mixed E1–E3)
Mechanical and interfacial: bulk mechanical	Loss of stiffness, recovery, creep resistance, fatigue resistance, or load-bearing capacity	Adjust network design, reinforcement, conditioning, or permitted loading	Greater stiffness may reduce stress relaxation, cell spreading, or matrix remodeling	[34,36] (E1)
Mechanical and interfacial: interface	Separation, cracking, sliding, or inadequate local load transfer	Adjust the curing sequence, introduce compatible interfacial chemistry, or add mechanical interlocking	Stronger bonding may increase local stiffness, chemical exposure, or stress concentration	[40,41,42] (E3; indirect)
Transport and cell distribution	Channel narrowing, increased flow resistance, inadequate oxygenation, regional cell loss, or uneven matrix distribution	Adjust channel geometry, construct thickness, swelling, degradation, or perfusion conditions	Greater porosity or perfusion may reduce mechanical integrity or increase flow-induced stress	[44,45] (mixed E1–E3)
Biofunctional	Intended tissue formation, phenotype, fusion, or bioactive delivery is not achieved	Match degradation to tissue formation and adjust cell density, biochemical induction, or bioactive delivery	Slower degradation, stronger reinforcement, or excessive retention may restrict remodeling, fusion, or effective dosing	[28,48,49] (E1)

Evidence linkage and reporting note: E1–E5 are defined in Section 1.3. The citations in Table 1 support representative mechanisms or design strategies rather than validated acceptance limits. Quantitative reporting should pair each value with the test definition, unit, reference state, and assessment time. Examples include dimensions in mm or percent change, mechanical quantities in kPa or MPa with the stated modulus or viscoelastic definition, interface strength, peel force, or fracture energy in test-appropriate units, transport parameters such as diffusivity, permeability, pressure drop, oxygen concentration, and flow rate in physical units, and biological outputs with a stated normalization denominator.

## 4. Use-Point Assessment with Sequential Use Points and Function-Specific Modules

### 4.1. Framework Structure

A single universal reference condition is not appropriate. Four reference states are distinguished: the precursor bioink or pre-print cell condition, the as-deposited construct, the post-crosslinking construct, and the first equilibrated wet-state construct. The pre-print state is used to identify printing-associated cellular or chemical effects. The digital design and as-deposited state define deposition accuracy. The post-crosslinking state defines immediate network establishment. The equilibrated wet state is generally the reference for subsequent longitudinal structural, mechanical, and transport comparisons. For operational use, equilibration should be declared prospectively, for example, when two consecutive measurements of mass, external dimensions, or another system-relevant wet-state attribute fall within a predefined tolerance; a fixed elapsed time alone does not establish equilibrium. Persistent defects already present after deposition or crosslinking are recorded as printing-origin conditions and are not normalized away by later comparison. A direct printed-versus-unprinted comparison also showed that metabolic activity and viability can provide complementary information after extrusion, supporting retention of a pre-print or unprinted biological comparator when printing-related cell effects are under study [50].

Three application-dependent use points are considered. The first is after stabilization and transfer. The second is during or at the end of culture or conditioning. The third is before implantation or another intended application. These use points need not be three distinct chronological tests: when the end-of-culture state is also the pre-implantation or pre-use state, the second and third use points may be combined. At each applicable use point, the construct is compared with the relevant reference condition and with predefined requirements.

Measurements should be selected according to the relevant failure domains defined in Section 3 and Table 1. The corresponding evaluation modules use the same names: Structural and dimensional, Mechanical and interfacial, Transport and cell distribution, and Biofunctional. Not every measurement is required for every construct. The assessment should focus on the requirements that are essential for the intended application.

If a criterion is not met, the earliest observed critical deviation should be identified within the available assessment resolution, the probable cause should be addressed only when supported by evidence, and the construct should be reassessed at the same use point. Table 2 summarizes representative measurements and actions at each stage.

**Table 2:** Sequential use-point assessment of post-printing maturation in cell-laden extrusion-printed hydrogel constructs.

Use Point	Main Question	Candidate Post-Printing Measurements	Action if Criteria Are Not Met	Representative Evidence
After stabilization and transfer	Did stabilization or handling cause a critical change from the reference condition?	Hydrated dimensions, pore or channel retention, handling integrity, interlayer continuity, and regional viability relative to the as-deposited and post-crosslinking reference states	Revise temporary support, crosslinking, environmental exposure, or handling	[16,17] (E1–E2)
During or after culture or conditioning	Does the construct remain structurally, mechanically, and biologically suitable during maturation?	Swelling, degradation, contraction, wet-state mechanics, interface integrity, channel retention, spatial viability, and tissue-specific matrix formation relative to the first equilibrated wet-state reference	Address the earliest deviation in Structural and dimensional, Mechanical and interfacial, Transport and cell distribution, or Biofunctional criteria	[28,32,45,48] (E1)
Before implantation or application	Does the construct meet its essential requirements after the complete pre-use pathway?	Geometry, wet integrity, load response when relevant, spatial cell status, intended biological function, retained bioactivity, and applicable safety-gate measurements after the complete pre-use pathway	Revise maturation, storage, transport, preparation, or final handling	[39,45,49] (mixed E1–E3)

#### Operational Decision Rules

Acceptance criteria should be defined prospectively for each essential attribute and use point. A performance target is the desired value or range. An acceptance limit is the boundary used for a decision. An optional warning band is an internal range that triggers additional observation before an acceptance limit is crossed. Limits should be justified by intended function, a biological or clinical target when available, engineering constraints, assay precision, measurement uncertainty, and normal batch variability. Statistical significance from a reference state does not by itself define failure, and absence of statistical significance does not establish acceptability.

Pass indicates that all essential attributes remain within their predeclared limits after accounting for measurement uncertainty. Conditional indicates that no essential limit is demonstrably violated but one or more results fall within a warning band, overlap an acceptance limit because of uncertainty, or require an additional measurement before progression. Fail indicates that at least one non-negotiable essential criterion is outside its acceptance limit. A safety-gate failure defined in Section 3.5 overrides performance-domain pass status. Criteria should be specified before testing together with units, reference state, sampling interval, replicate structure, and the action associated with each decision.

The use-point assessment plan should be reported as a matrix that identifies the reference condition, measurement set, interpretation rule, and action for each applicable use point and module. A vascularized osteochondral construct, for example, may require a culture use point together with the Transport and cell distribution module and the Mechanical and interfacial module. When an essential criterion is not met, progression should pause until the earliest observed critical deviation has been addressed and the construct has been reassessed at the same use point.

### 4.2. Application-Specific Use Points and Critical Requirements

The relevant use point is determined by the function required at the next stage of use. The same post-printing change may therefore be acceptable in one application but critical in another. Table 3 presents representative examples. The listed requirements are illustrative and should be defined for the individual construct and intended application. The requirements in Table 3 are interpreted using the same four evaluation modules defined in Section 3, Table 1, and Section 4.1.

**Table 3:** Representative use points and illustrative post-printing requirements for selected bioprinted constructs.

Construct Type or Application	Representative Use Point	Essential Requirements	Main Post-Printing Concern	Representative Evidence
Cartilage	End of a defined chondrogenic culture period or before downstream testing	Hydrated geometry, wet-state mechanical response, chondrocyte viability, phenotype maintenance, and cartilage matrix formation	Geometry or viability remains within its predefined limit, but phenotype or cartilage-matrix measures fall outside the predefined target range	[29] (E1)
Bone	End of a defined osteogenic culture or conditioning period	Mineral volume and density, construct stiffness, cell viability, osteogenic marker expression, and cellular organization	Mineral volume or density, mechanical response, or osteogenic marker panel remains outside the predefined target range at the use point	[54] (E1)
Full-thickness skin model	After the defined air–liquid interface maturation period and before functional testing	Layer organization, epidermal differentiation, ECM organization, barrier integrity, and hypodermis-related gene expression	Layer organization or barrier-function measures remain outside the predefined target range after air–liquid interface maturation	[55] (E1)
Perfusable thick construct	After flow initiation and during the defined perfusion period	Channel continuity, internal oxygenation, perfusion continuity, and regional cell viability	Channel geometry remains within tolerance but oxygen or perfusion metrics fall outside the predefined target range	[46] (E1)

Table 3 presents example attributes rather than validated specifications. For a specific construct, qualitative terms such as preserved, sufficient, or acceptable should be replaced before testing by measurable limits with units, the applicable reference state, the assessment time, and the uncertainty rule.

The following cartilage example shows how these application-specific requirements can be translated into a sequential assessment workflow.

### 4.3. Illustrative Application to a Cartilage Construct

**Figure 3:**
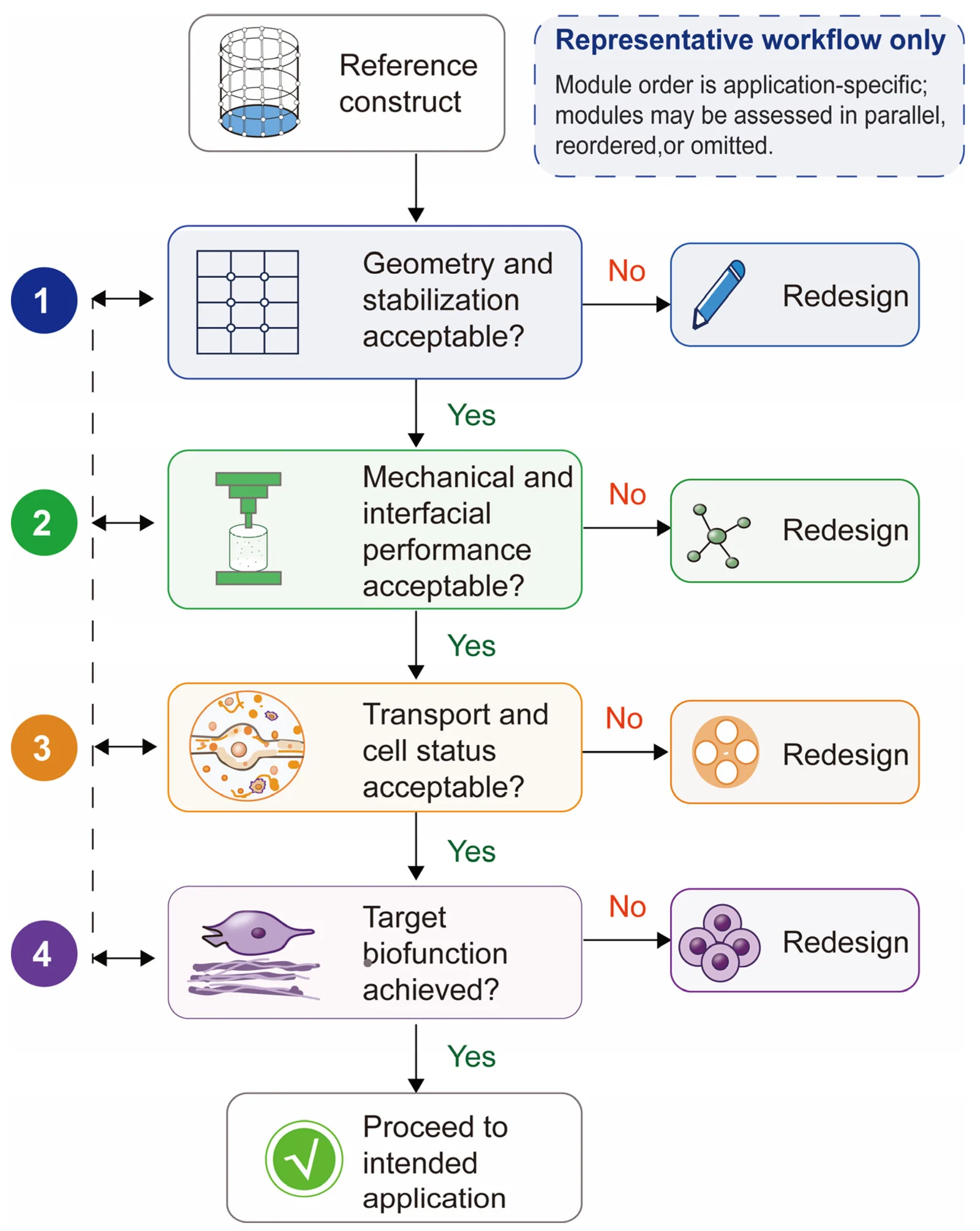
Illustrative sequential use-point assessment for a cartilage construct. Module order is not universal. Application-specific modules may be applied in parallel, reordered, or omitted. Cross-domain effects should be reassessed after redesign.

The framework can be illustrated using a cell-laden extrusion-printed hydrogel construct intended for cartilage maturation. The target use point is the end of a defined chondrogenic culture period before implantation or another downstream application. The example is not restricted to one bioink and does not prescribe universal measurements or acceptance limits. The sequential assessment workflow is shown in Figure 3.

#### 4.3.1. Early Post-Printing Assessment

For this cartilage example, the pre-print cell condition is retained to assess printing-related cell injury, the digital design and as-deposited construct are used to evaluate deposition accuracy, and the post-crosslinking and first equilibrated wet-state constructs are used for later structural and mechanical comparisons. External dimensions, filament and pore geometry, layer alignment, spatial cell distribution, and viability are recorded at the applicable reference states. After the complete crosslinking and handling sequence, measurements are repeated to determine whether stabilization has introduced or worsened a critical deviation. Day 0 viability and spatial cell distribution have been used as reference measurements in extrusion-printed mesenchymal stem cell (MSC)-laden cartilage constructs, while direct comparison of pre-crosslinked and post-crosslinked chondrocyte-laden alginate constructs has shown that subsequent stabilization can affect mechanics, degradation, and cell response [23,56]. A critical deviation at this stage indicates that the deposition, crosslinking, or handling procedure should be revised before long-term culture.

#### 4.3.2. Culture and Target Use-Point Assessment

During chondrogenic culture, longitudinal measurements should cover dimensional change, mass retention, degradation, contraction, wet-state mechanics, spatial viability, cell content, and cartilage matrix formation. Degradation should be interpreted together with tissue development. In extrusion-printed methacrylated guar gum bioinks, a higher methacrylation degree reduced long-term mass loss and improved structural stability, while the more stable formulation increased GAG production and collagen type II alpha 1 chain (COL2A1) expression in bone marrow-derived MSC-laden constructs [57]. In cell-laden methacrylated flaxseed gum constructs, the printed hydrogel maintained structural integrity and exhibited a degradation period of approximately 66 days while supporting stem cell chondrogenesis and subsequent cartilage formation, illustrating the importance of matching material persistence with tissue maturation [58].

At the target use point, assessment should include the mechanical and biological requirements relevant to cartilage. Candidate mechanical measurements include compressive modulus with a stated definition, stress relaxation, and recovery under the relevant wet-state conditions. Biological assessment should use an application-specific panel rather than a single marker and may include spatial GAG content, type II collagen or aggrecan deposition, collagen I and X or other fibrocartilaginous and hypertrophic markers, cell content-normalized matrix production, and histological organization. Spatial measurements are needed when whole-construct values may conceal regional loss of viability or matrix formation.

#### 4.3.3. Decision and Redesign

If an essential requirement is not met, redesign should address the earliest observed critical deviation that can be resolved from the assessment schedule, and the revised construct should be tested again under the same maturation conditions. Because a change made for one domain may alter another domain, all affected essential attributes should be reassessed.

### 4.4. Validation Status and Scope

This framework is conceptual, research-oriented, and has not been prospectively validated. It should not be interpreted as a universal specification system or regulatory qualification strategy. Future studies should determine whether it can consistently identify clinically or experimentally meaningful post-printing changes and support reproducible decisions across different bioinks, cell sources, construct designs, laboratories, and applications.

## 5. Outlook for Standardization, Prediction, and Translation

Progress will depend on tracking how cell-laden extrusion-printed hydrogel constructs evolve after deposition, predicting their later state, and determining whether they meet specified use-point requirements. Three priorities are central. They are standardized longitudinal characterization, prediction of maturation trajectories, and translation-oriented development of product-specific specifications.

### 5.1. Standardized Longitudinal Characterization

Longitudinal studies should distinguish the freshly printed, immediately stabilized, environmentally equilibrated, and specified use-point conditions. Additional time points may be required during extended remodeling. Each measurement should be linked to a defined exposure history.

Reporting should include crosslinking sequence and dose, temperature, medium composition, ionic or enzymatic exposure, incubation duration, medium exchange, perfusion, loading, and handling. These variables can change maturation even when formulation is unchanged. Stage-specific reports should identify the conditions that produced each measurement.

Controls should match the question. Molded gels help isolate material-level behavior, whereas acellular extrusion prints help distinguish architectural and interfacial effects. Direct biological use-point assessment requires cell-laden extrusion-printed constructs tested under the relevant maturation conditions. Batch reproducibility should also be assessed in cell-laden printed constructs, especially for natural polymers, multistep crosslinking, and cell-containing formulations. To permit evidence-quality judgment, studies should report the numbers of independent bioink preparations, printed constructs, and biological replicates, the spatial sampling strategy, all specified time points, missing or excluded samples, and whether the main result was reproduced in an independent batch or experimental condition.

Process monitoring can establish the initial fabrication state needed to interpret later changes. During embedded extrusion bioprinting, integrated microscopy and computer vision have been used to quantify deposited area and positional accuracy relative to the intended design [13]. But such measurements do not determine how the construct subsequently changes during culture. Post-printing monitoring therefore requires complementary measurements of the maturation environment and construct response. In centimeter-scale fibroblast-laden gelatin–alginate–fibrinogen hydrogel constructs, online monitoring of temperature, pH, and oxygen was combined with Raman-based lactate analysis and magnetic resonance imaging [45]. Imaging of the same construct immediately after printing and after 16 days of culture revealed pore occlusion, shape deformation, and localized matrix degradation. This study illustrates how environmental and metabolic monitoring can be linked to longitudinal structural changes in a cell-laden hydrogel construct under a defined culture history.

### 5.2. Predictive Modeling of Maturation Trajectories

Data-driven modeling in extrusion bioprinting currently focuses mainly on precursor rheology, formulation selection, and printability. Existing studies have identified rheological descriptors associated with printability, classified printable biomaterial formulations, and estimated hydrogel viscosity from composition and shear rate [59,60,61]. These approaches support initial material and process selection but do not predict how a cell-laden construct changes after printing.

Direct models of post-printing maturation remain limited and currently address specific biological or physical processes. A cellular automata model calibrated with 11-day observations of extrusion-bioprinted breast cancer cells in gelatin–alginate hydrogels predicted cell proliferation under different initial cell densities and bioink formulations [14]. A physics-based model of extrusion-bioprinted constructs coupled nutrient diffusion and consumption with cell viability and proliferation and was validated against experimental changes in cell viability [62]. A computational model of post-processing contraction in bioprinted bone constructs incorporated nonlocal cellular traction and evaluated how hydrogel geometry, mechanical properties, and gravity influence construct deformation [63].

These studies represent complementary approaches to post-printing modeling. They predict cellular behavior, couple transport with cell growth, or simulate contraction-driven construct deformation. However, each addresses only part of maturation. None integrates network degradation, dimensional change, mechanics, transport, cellular remodeling, and tissue-specific matrix formation into a complete construct trajectory.

The next step is therefore not simply to predict more isolated endpoints, but to model how maturation processes influence one another over time. The standardized longitudinal data described in Section 5.1 could be used to calibrate and test models that predict the sequence of changes, identify the probable earliest observed critical deviation, and estimate whether and when the construct will meet the requirements of a specified use point. Such predictions require validation against independent time points, constructs, or maturation conditions before they can support use-point decisions.

### 5.3. Translation-Oriented Assessment and Future Specification Strategies

For translational development, the framework in Section 4 should be viewed as a research-oriented evidence-organization scheme rather than a regulatory qualification strategy. The U.S. Food and Drug Administration (FDA) documents Potency Tests for Cellular and Gene Therapy Products (2011 final guidance) and Potency Assurance for Cellular and Gene Therapy Products (December 2023 draft guidance), together with the European Medicines Agency (EMA) Guideline on quality, non-clinical and clinical requirements for investigational advanced therapy medicinal products in clinical trials, are guidance documents with distinct legal and procedural status [64,65,66]. ISO/TS 21560:2020 is a technical specification for general requirements of tissue-engineered medical products, while the 2026 Joint Research Center report Putting Science into Standards–3D Bioprinting: Towards Standards in Biomedicine is a scientific standardization report. These two documents do not establish legally enforceable product-specific acceptance criteria [67,68]. None of these sources provides validated acceptance criteria specific to cell-laden extrusion-bioprinted hydrogel constructs.

The regulatory route for a particular construct depends on jurisdiction, composition, viable-cell source, device components, mechanism of action, and intended use. A cell-laden bioprinted product may therefore fall within cellular or biological, tissue-engineered or advanced therapy, device-integrated, or combination-product pathways. The proposed use-point framework does not determine this classification. At the research stage, it may instead help organize product-specific evidence for geometry, wet-state integrity, spatial cell status, intended function, manufacturing consistency, and stability during storage and handling. Destructive testing describes only the tested construct, so companion samples must be shown to represent the same batch and exposure history. Nondestructive methods have been used to estimate cell-generated contraction, assess mechanical properties by ultrasound elastography, and monitor metabolism and internal remodeling using physicochemical sensors, Raman analysis, and magnetic resonance imaging [32,39,45]. These methods are promising, but each must be validated against the relevant construct attribute. The next step is to identify a limited number of essential attributes and determine prospectively whether they are repeatable and predict the required construct outcome under the complete pre-use pathway.

### 5.4. Limitations of the Current Evidence and Proposed Framework

Studies differ in materials, cells, construct designs, maturation conditions, assessment methods, and reporting quality, which limits direct comparison. Longitudinal E1 evidence remains incomplete, particularly for cell-laden interfaces, quantitative transport, regional mechanics, retained bioactivity, and chemical or microbiological safety. Some mechanistic conclusions therefore rely on E3–E5 evidence from acellular printed constructs or bulk hydrogels and are identified as indirect support. The evidence-directness classification and use-point framework proposed in this review remain qualitative and require prospective validation across materials, laboratories, and applications.

## 6. Conclusions

Cell-laden extrusion-bioprinted hydrogel constructs should be evaluated beyond immediate printability because their structure, mechanics, transport, biological function, and safety-relevant state can continue to change after extrusion. Post-printing maturation may support tissue formation, but it may also cause structural, mechanical, interfacial, transport, or biofunctional failure. Direct longitudinal studies of cell-laden extrusion-printed constructs should therefore provide the main evidence, while acellular prints and bulk hydrogels should be used only to clarify relevant mechanisms.

Use-point assessment determines whether a construct meets prospectively defined, application-specific requirements at a specified stage after printing. It links multiple reference states and maturation history with the earliest observed critical deviation, targeted redesign, reassessment under the same conditions, and a cross-cutting safety gate. The proposed framework is a conceptual research structure rather than a validated specification system. Future studies should test whether it can support reproducible assessment across different materials, cells, construct designs, and applications.

## Data Availability

No new data were created or analyzed in this study. Data sharing is not applicable to this article.

## Appendix A

**Table A1:** Study-level evidence summary used to support the post-printing framework. NR indicates not reported or not available in the evidence extraction. E1-E5 and the C modifier are defined in Section 1.3.

Reference	Bioink/Crosslinking	Cell Model/Density	Printing/Architecture	Culture or Exposure/Time Points	Post-Printing Measurements	Observed Maturation or Failure Behavior	Evidence Class	Longitudinal/Same Construct	Replicates/Spatial Assessment
[1]	Gelatin–alginate; thermal/ionic	Acellular formulation/printability study	Extrusion; artifact/shape-fidelity geometries	Immediate post-print assessment	Rheology, extrusion, filament/shape fidelity	Defines a printability window; no direct biological maturation trajectory	E3	No	NR/whole construct
[2]	Multiple hydrogel bioinks; formulation dependent	Acellular formulation/shape-fidelity study	Extrusion; standardized shape-fidelity artifacts	Immediate	Filament spreading, collapse, geometry	Quantifies early shape fidelity	E3	No	NR/whole construct
[3]	GelMA-based biomaterial; photocrosslinking	NIH/3T3 fibroblasts; 5 × 10^6^ cells/mL	Direct-write extrusion; 3D tissue geometry	Days 1, 7, 14, and 21	µCT filament/pore geometry, mechanical properties, degradation, Live/Dead viability	Printed constructs maintained 71–77% viability and consistent mechanical properties over 21 d while geometry and degradation were tracked	E1	Yes/repeated groups at four time points	n = 3/whole-construct and spatial imaging
[4]	Photocurable collagen vs. GelMA; light crosslinking	Cell-laden; density NR	DLP and extrusion comparison	Up to 7 d	Printability, viability, spreading, proliferation, gene expression	Collagen and GelMA differ in printing and cell response	E1	Yes/repeated groups	NR
[5]	Stress-relaxing hydrogels; covalent network variants	Stem cells; density NR	Bulk 3D hydrogel	Days	Stress relaxation, spreading, fate/activity	Relaxation regulates cell behavior independent of initial modulus	E4	Yes/repeated groups	Replicates reported; bulk
[6]	Soft tissue bioinks; crosslinking system-specific	Cell-laden	Extrusion 3D scaffolds	0–7 d	Nondestructive mechanics, swelling/material loss, cell proliferation	Whole-construct mechanics evolve during culture	E1	Yes/same construct supported	Reported; whole construct
[7]	Gelatin-HA-genipin	High cell density; specific density NR here	Extrusion	Cell assessment on days 1 and 3; formulation disintegration over 48 h	Shape stability, degradation/disintegration, viability	Cell viability increased from day 1 to day 3; one formulation fully dissolved within 48 h at 37 °C while others partially disintegrated	E1	Yes/repeated groups; disintegration tested separately	NR
[8]	Thiol-norbornene gelatin; photopolymerization	NHDF; 2.5 × 10^6^ cells/mL	Extrusion/photocuring	Days 1, 7, and 14 post-printing; proliferation to 21 d	Cure kinetics, mechanics, printability, post-print viability and proliferation	Rapid curing supported high post-print viability and longer-term cell spreading/proliferation; response was dose dependent	E1	Yes/repeated groups	n = 3/spatial confocal imaging
[9]	GelMA; photoinitiator-dependent photocrosslinking	Acellular mechanistic network study	Bulk hydrogel	Seconds–minutes	Reaction kinetics, real-time modulus	Photoinitiator chemistry and oxygen influence network formation	E5	Real-time repeated	NR
[10]	Gelatin-HA dynamic + photocrosslinked double network	BMSCs seeded onto a printed acellular scaffold; not cell-laden during extrusion	3D extrusion	Days 1, 3, and 5 after seeding	Self-healing, printability, mechanics, post-seeding viability and proliferation	The double network supported recovery and printed stability; biological compatibility was evaluated after scaffold seeding	E3	Repeated groups after seeding; not a cell-laden printing trajectory	NR
[11]	Bioink formulations; crosslinking varies	Acellular/printability focus	Extrusion artifact quantification	Immediate	Rheology and print artifact metrics	Links rheology with early print quality	E3	No	NR
[12]	Hydrogel inks; formulation varies	Acellular/printability focus	Extrusion	Immediate	Optical geometry and modeling	Improves quantitative early quality assessment	E3	No	Regional optical analysis
[13]	Embedded-printing inks/support bath	Cardiac fibroblasts; 2 × 10^6^ cells/mL in the validation experiment	Embedded extrusion	During deposition; cell viability at 24 h	Integrated microscopy, deposited area, positional accuracy, cell distribution and viability validation	In-process defects were quantified relative to design; cell-laden validation confirmed imaging and short-term viability compatibility	E2	In-process repeated imaging; 24 h cell endpoint	Spatial image analysis
[14]	Gelatin–alginate	Breast cancer cells; density/formulations varied	Extrusion 3D hydrogel structures	Up to 11 d	Cell counts/proliferation + cellular automata model	Post-print cell proliferation depends on formulation and initial density	E1 + C	Yes/repeated time points	Reported; spatial/model outputs
[16]	GelMA; reversible physical + UV stabilization	Human chondrocytes; density NR	Extrusion cartilage-oriented constructs	Days 1, 7, and 28	Printability, viability, proliferation, stabilization response	Physical pre-stabilization improves deposition before permanent curing	E1	Yes/repeated groups	NR
[17]	Methylcellulose/GelMA; thermal support + photocrosslink	Human primary osteoblast-laden print used for biological validation; density NR	Extrusion	Cell viability at 48 h; acellular swelling/degradation up to 60 d	Shape integrity, swelling/degradation, printed-cell viability	Temporary methylcellulose support improved deposition, persistent GelMA stabilization maintained the structure, and a cell-laden printed lattice retained high short-term viability	E2	Cell endpoint plus separate acellular time course	NR
[18]	Hyaluronan; enzymatic pre-gel + visible-light crosslink	hMSCs; 1–5 × 10^6^ cells/mL	Extrusion	24 h and 14 d	Rheology, printability, reinforcement, printed-cell viability	Pre-gel supports extrusion; light exposure reinforces filament	E1	Yes/repeated groups	NR
[19]	GelMA; photo + microbial transglutaminase dual crosslink	Acellular extrusion-printed grids; cell-laden micropatterns made by photolithography	Extrusion printing and photolithography	Post-print treatment; 1 d cell endpoint in micropatterns	Stiffness, swelling, geometry	Secondary crosslinking increases stiffness and geometric stability	E3	Treatment comparison; no cell-laden extrusion trajectory	NR
[20]	HA single/double network; dynamic covalent + permanent network	Cell-laden; density NR	Extrusion	Immediate/short term	Recovery, mechanics, printability, viability	Dynamic bonds support extrusion; second network improves stability	E2	No	NR
[21]	Dynamic hydrogel bioinks; small-molecule bond modulators	Cell-laden; density NR	Extrusion	Immediate/short term	Bond exchange, printability, mechanics, cell compatibility	Small molecules tune dynamic crosslinking and printed stability	E2	No	NR
[22]	Alginate–gelatin; Ca^2+^ post-print treatment	Acellular	Extrusion scaffolds	Post-print swelling/degradation	Swelling, degradation, geometry	Post-print ionic treatment changes swelling and lifetime	E3	Time dependent	NR
[23]	Alginate with alternative cationic pre-crosslinkers	Chondrocyte/cell-laden cartilaginous tissues; density NR	Extrusion cartilage constructs	Culture time points NR here	Mechanics, degradation, cell response, matrix accumulation	Cation choice alters post-print mechanics and tissue response	E1	Yes	NR
[24]	HA-collagen stress-relaxing hydrogels	Cells in 3D bulk culture; density NR	Bulk hydrogel	Days	Cell spreading, fiber remodeling, focal adhesions	Faster relaxation supports remodeling	E4	Yes	NR
[27]	Collagen inks; physical assembly vs. bioorthogonal covalent crosslink	Cell-laden	Embedded extrusion in microgel bath	Long-term culture	Microstructure, cell spreading, contraction/shape retention	Covalent crosslinking reduces cell-induced contraction	E1	Yes	Spatial microstructure
[28]	Rapidly degrading oxidized-alginate-type bioinks	Cartilage microtissues	Extrusion/biofabrication	Long-term cartilage culture	Degradation, fusion, viability, GAG-rich matrix	Rapid degradation can support microtissue fusion and matrix formation	E1	Yes	Reported; tissue distribution assessed
[29]	Gelatin–alginate; composition/stiffness varied	Cell-loaded cartilage constructs	Extrusion	Long-term culture	Morphology, mechanics, viability, cartilage markers	Optimized composition preserves shape while supporting collagen II	E1	Yes	NR
[30]	GelMA–gelatin-hydroxyapatite	Osteoblast-laden	Extrusion composite hydrogel	Days 1, 7, 14, and 28	Swelling/degradation, cell viability/proliferation, osteogenic differentiation and mineralization	Hydroxyapatite reduces swelling/degradation and promotes osteogenesis	E1	Yes/repeated groups	n = 5 for reported culture/degradation measures/whole construct
[31]	GelMA–alginate; ionic + covalent crosslink	Sheep adipose-derived stem cells (sADSCs); density NR	Extrusion	Cell viability at 24 h and 7 d; acellular swelling/degradation to 14 d	Viability, proliferation, swelling, degradation, mechanics	Composition/crosslinking tune wet-state properties	E1	Yes/repeated groups	NR
[32]	Bioprinted hydrogel under cell traction	Contractile cells; density NR	Bioprinted construct with force measurement	Culture over time	Nondestructive macroscopic tension/contractility	Cell-generated contraction can be tracked longitudinally	E1	Yes/same construct compatible	Whole-construct force
[33]	Shrink-resistant collagen-HA composite	Cell-laden	3D bioprinting	Long-term culture	Shrinkage, geometry, cell response	Network design improves dimensional retention against cell contraction	E1	Yes	Spatial/whole construct
[34]	Self-healing tough hydrogel bioink	Cell-laden	Direct extrusion	Days 1, 3, 7, and 14	Toughness, cyclic/fatigue resistance, cell compatibility	Self-healing network improves antifatigue performance	E1	Yes/repeated groups	Replicates NR
[35]	Elastin-containing bioactive double network	Cell-laden	Extrusion	Days 1 and 7 in vitro; additional regeneration endpoints	Mechanical reinforcement, tissue regeneration outcomes	Double network supports tough elastic constructs	E1	Yes/repeated groups	NR
[36]	Composite hydrogel + human bone allograft particles	hMSCs; approximately 3 × 10^6^ cells/mL	Extrusion	Days 1, 7, 14, 21, and 28	Stiffness, swelling, viability, migration, ALP and osteogenic markers	Allograft particles increase stiffness and support osteogenesis	E1	Yes/repeated groups	NR
[40]	Dual-network hydrogel + supramolecular gelator	Acellular	3D extrusion; layer interfaces	Post-processing curing	Interlayer adhesion/tensile performance	Simultaneous curing improves continuity vs. layerwise curing	E3	No	Interface-level tests
[41]	Hydrogel-elastomer chemically coupled interface	Acellular	Multimaterial printing	Post-print mechanical tests	Interfacial adhesion	Chemical coupling strengthens soft-material interfaces	E3	No	Local interface
[42]	Hydrogel-PLA mechanically interlocked interface	Acellular	3D-printed soft–hard interface	Mechanical testing	Interfacial strength, toughness, failure location	Mechanical interlocking improves strength; failure may remain in hydrogel	E3	No	Local interface
[44]	Perfused hydrogel vascular model	Acellular printed hydrogel subsequently endothelialized with HUVECs	3D-printed perfusable hydrogel channel	Post-print endothelialization and perfusion exposure	Permeability, endothelial barrier, leakage	Printed channel geometry enabled perfusion, while endothelial barrier function required direct post-seeding measurement	E3	Repeated perfusion measurements; not cell-laden extrusion	Regional/channel level
[45]	Gelatin–alginate–fibrinogen	Fibroblast-laden; centimeter scale	Extrusion + bioreactor	0–16 d	pH, O2, lactate/Raman, MRI, morphology, perfusion	Pore occlusion, deformation and localized degradation develop during culture	E1	Yes/same construct imaging	Multimodal regional monitoring
[48]	Alginate-based bioinks with tuned degradation	Cartilage cells/microtissues; density NR	Extrusion	Long-term cartilage culture	Degradation, GAG/tissue formation, fusion	Degradation rate must match tissue formation	E1	Yes	NR
[49]	Proteinaceous scaffold with TGF-β1 + FGF-18 delivery	Cell-containing cartilage construct	3D bioprinting	Cell-laden culture to 21 d; in vivo regeneration endpoints	Growth-factor release, chondrogenesis, cartilage repair	Controlled dual-factor delivery promotes chondrogenesis/regeneration	E1	Yes/repeated groups and in vivo endpoint	NR
[39]	Suspended bioprinting hydrogel	Acellular suspended-printing evidence	Suspended bioprinting	In situ during/after print	Ultrasound shear-wave elasticity	Nondestructive elasticity monitoring is feasible	E3	Yes/same construct compatible	Whole/local elasticity mapping
[54]	Bone-derived cell-laden hydrogel scaffold	Human bone-derived cells; physiological densities compared	Extrusion	Dynamic loading culture	Mineralization rate, stiffness, cell organization	Cell density influences matrix mineralization and stiffness maturation	E1	Yes	Reported; spatial organization
[55]	Full-thickness skin bioink system	Skin cell populations including hypodermal component; density NR	Bioprinted multilayer skin model	Days 5 and 10 of skin differentiation	Layer organization, gene expression, differentiation/barrier-related outcomes	Hypodermis alters maturation-related gene expression	E1	Yes/repeated groups	Spatial layers
[46]	Cell-laden thick constructs with perfusable microchannels	Cell-laden; density NR	3D-bioprinted thick channel network	Perfusion/culture	Internal oxygen levels, channel network	Direct O2 measurements reveal transport state inside thick constructs	E1	Yes	Spatial oxygen measurements
[56]	Alginate-based in situ crosslinking cartilage system	Primary juvenile bovine MSCs; 20 × 10^6^ cells/mL	Extrusion with in situ crosslinking	Days 0, 3, and 7 for viability/distribution; chondrogenic culture to 56 d	Viability, spatial cell distribution, mechanics/degradation, GAG, collagen and gene expression	In situ crosslinking maintained viability and spatial distribution and supported long-term chondrogenic maturation	E1	Yes/repeated groups	n ≥ 3/top, middle and bottom spatial assessment
[57]	Methacrylated guar gum; photocrosslinking	Bone-marrow MSC-laden; density NR	Extrusion cartilage constructs	Long-term culture	Mass loss, structural stability, GAG, COL2A1	Higher methacrylation reduces mass loss and supports chondrogenic output	E1	Yes	NR
[58]	Methacrylated flaxseed gum; photocrosslinking	Stem cell-laden; density NR	Extrusion	Up to ~66 d degradation/cartilage formation	Integrity, degradation, chondrogenesis	Material persistence can be matched to cartilage maturation	E1	Yes	NR
[59]	Rheology-modified hydrogel formulation library	Acellular formulation data	Printability/process study	Immediate	Rheological descriptors + machine learning	Rheology features predict printability, not post-print maturation	E3 + C	No	Dataset-level
[60]	Biomaterial formulation library	Acellular/process data	Direct ink writing/printability	Immediate	Formulation descriptors + machine learning	Predicts printable formulations, not maturation	E3 + C	No	Dataset-level
[61]	Hybrid hydrogel bioink; formulation-dependent	Acellular/process characterization focus	Extrusion-based printing	Immediate	Rheology, printability, ML property prediction	Predicts precursor/printing properties rather than maturation	E3 + C	No	Dataset-level
[62]	Cell-laden extrusion construct model	Cell viability/proliferation experimental validation	Extrusion-based constructs	Tissue maturation time course	Nutrient diffusion/consumption, viability, proliferation	Model couples transport with cell growth and matches experimental viability change	E1 + C	Yes	Model + experimental validation
[63]	Bioprinted bone contraction model	Cellular traction represented computationally	Bioprinted bone geometry	Post-processing	Contraction, geometry, mechanics, gravity effects	Nonlocal traction predicts deformation and sensitivity to construct properties	C	Modeled time course	Computational
[50]	Alginate–gelatin; thermal + CaCl2 crosslinking	MS5 stromal cells; 4 × 10^5^ cells/mL	Extrusion; structures up to 10.3 ± 1.4 mm	0, 3 h, 24 h, 72 h, 7 d	Live/dead, ATP, morphology; printed vs. unprinted controls	Live/dead and metabolic activity are complementary and may diverge	E1	Yes; repeated time points	N = 3–4+ depending formulation; whole/sample imaging
[52]	GelMA; type I/II photoinitiators	MSC-encapsulated; density NR	Cell-laden hydrogel; light-assisted bioprinting relevance	Up to 1 week	Elastic modulus, ROS, pore size, viability	Higher PI can increase modulus while increasing ROS and reducing viability	E4	Yes	Replicates reported; bulk/cell-laden
[53]	Gelatin/GelMA materials with different endotoxin burden	Macrophage-cancer cell 3D model; density NR	Three-dimensional printed in vitro tumor model	Days 1, 4, and 7 post-bioprinting; therapy exposure	Inflammatory response, cell interaction, therapeutic response	Endotoxin changes immune behavior and apparent treatment effect	E1	Yes/repeated groups	Three-dimensional model; regional/cellular readouts
[15]	Alginate, collagen, and Pluronic inks; formulation-dependent extrusion behavior	Acellular process-control study	Extrusion bioprinting with integrated camera, convolutional neural-network classification, and closed-loop correction; single-line and infill patterns	Real-time during printing; extrusion errors corrected within approximately 10 s	Image-based classification of good, over-, and under-extrusion; real-time monitoring; automated parameter correction	Closed-loop control detected and corrected extrusion errors across inks with different rheological properties, supporting process-state quality control rather than post-printing maturation assessment	E3	Real-time process monitoring; not a post-printing longitudinal study	Multiple real and synthetic training/testing datasets; process-level image assessment
[43]	PVA/alginate/gellan-gum sensor hydrogel with conductive fillers; cell-laden Matrigel/collagen bioink	C2C12 myoblasts; 1 × 10^6^ cells/mL	Multimaterial extrusion bioprinting; centimeter-scale biphasic sensorized muscle construct	Immediately after printing and after 10 d tissue development	Spatial Live/Dead, interface imaging/histology, shape retention, differentiation, mechanoelectrical response	Bioprinted constructs maintained high post-printing viability and a coherent sensor–tissue interface through 10 d of maturation	E1	Yes; interface followed through 10 d	Three experimental replicates with technical replicates for viability; n = 3 for mechanical/sensing tests
[47]	GelMA/gelatin colloidal bioink with oxygen-generating CaO_2_/MnO_2_-containing microparticles	Human dermal fibroblasts and hMSCs; density NR	Extrusion-based cell-laden printing; multilayer and tissue-mimetic constructs	Oxygen release to 10 d; cell assessment to 7 d; HIF1-α at 18 h	Oxygen/H_2_O_2_ release, rheology, printability, mechanics, degradation, Live/Dead, HIF1-α	Oxygen availability was directly tunable and measurable while maintaining extrusion printability and cytocompatibility	E1	Yes/repeated groups for oxygen release and biological endpoints	Mostly n = 3; whole-construct and local imaging reported
[51]	Methacrylated collagen peptide with short collagen-I microfibers and xanthan gum; UV curing	Human MSCs; density NR	Cell-laden bioprinted fibrous collagen-derived constructs	Culture and differentiation to 28 d	Cell spreading, metabolic activity, extracellular matrix, COL-I, scleraxis, differentiation	Fibrous constructs supported increasing metabolic activity, matrix production, and lineage-related protein expression during 28 d	E1	Yes; maturation assessed over 28 d	Replicate number NR in abstract; cellular and matrix readouts reported
[25]	GelMA/PEO shear-oriented bioink; UV photocrosslinking; carrageenan support bath	C2C12, HUVECs, BMSCs, and MC-3T3 cells; densities NR here	Embedded extrusion; freeform anisotropic, vascular, and muscle-patch structures	Day 1 viability; cell morphology/spreading after 3 and 7 d	Filament geometry, anisotropy, Live/Dead, cytoskeletal orientation, immunofluorescence	Extrusion-induced alignment enabled viable anisotropic constructs and guided cell spreading/orientation	E1	Repeated groups at 1, 3, and 7 d/same-construct tracking NR	Replicates NR here/spatial fluorescence imaging
[26]	Dilute/concentrated PEGDA streams with PAA microgels; photopolymerization	Acellular material-processing study	Millifluidic layer multiplication followed by filament 3D printing	Immediate post-fabrication characterization and programmed actuation	Rheology, internal architecture, swelling, mechanical contrast, and shape transformation	Hierarchically patterned crosslink density produced seamless filaments with programmable mechanical contrast	E3	No longitudinal biological study	Replicates NR here/multiscale structural assessment
[37]	TMSC-laden collagen bioink reinforced with chiral or chevron PCL patterns	Tonsil-derived mesenchymal stem cells; density NR here	Multimaterial 3D printing of hybrid biopatches; porcine mucosal-defect application	Cell assessment on days 1 and 5; mechanical/fatigue testing; in vivo endpoint	Live/Dead, proliferation, contraction, tensile/fatigue behavior, histology, epithelial markers	Pattern geometry controlled anisotropy and reinforcement while maintaining high viability; chiral patches improved mucosal repair	E1	Repeated in vitro time points and an in vivo endpoint/same-construct tracking NR	n = 4 reported for experiments/whole-patch and histological spatial assessment
[38]	Salting-out-tuned photocrosslinkable hydrogel ink	Acellular; synthetic vessel training model	Direct ink writing of multilayer hydrogel structures and synthetic blood vessels	Immediate/post-cure mechanical and surgical-training assessment	Rheology, print width, modulus, tensile/hoop response, leakage, electrocautery, and anastomosis	Salting-out enabled printable hydrogels spanning 0.193–1.072 MPa and mechanically tunable vessel models	E3	No biological maturation trajectory	n = 150 print-width observations per ink; other replicates as reported/whole construct

**Table A2:** Representative overlapping time scales of post-printing processes. These ranges indicate dominant process regimes and are not universal stage boundaries.

Process Class	Representative Dominant Time Scale	Key Rate-Controlling Factors	Representative Evidence
Flow recovery and thermoreversible stabilization	Seconds to minutes	Shear recovery, temperature, polymer concentration, reversible physical interactions	[16,17]
Ionic or photochemical stabilization	Seconds to hours, with later equilibration possible	Ion diffusion, light dose, photoinitiator chemistry, oxygen, optical path length, construct thickness	[8,9,22,23,52]
Enzymatic or dynamic-covalent network evolution	Minutes to days	Enzyme activity, functional-group concentration, bond-exchange kinetics, temperature and medium	[18,19,20,21]
Hydration, ion exchange and soluble-fraction equilibration	Minutes to days	Medium composition, ionic strength, geometry, diffusion distance and network density	[22,23,31]
Degradation, stress relaxation, cell-driven contraction and ECM deposition	Hours to weeks or longer	Network lability, cell density/activity, tissue formation, loading and culture history	[5,24,27,28,29,30,31,32,33]

**Table A3:** Database-specific literature-search strings used for the revision.

Database	Complete Search String
PubMed	((bioprint* [Title/Abstract]) OR (“extrusion bioprinting” [Title/Abstract]) OR (“embedded bioprinting” [Title/Abstract]) OR (“coaxial bioprinting” [Title/Abstract])) AND ((bioink* [Title/Abstract]) OR (“cell-laden hydrogel” [Title/Abstract]) OR (“cell laden hydrogel” [Title/Abstract])) AND ((“post-printing” [Title/Abstract]) OR maturation [Title/Abstract] OR culture [Title/Abstract] OR crosslink* [Title/Abstract] OR swelling [Title/Abstract] OR degradation [Title/Abstract] OR contraction [Title/Abstract] OR “stress relaxation” [Title/Abstract] OR mechanic* [Title/Abstract] OR interface* [Title/Abstract] OR transport [Title/Abstract] OR perfusion [Title/Abstract] OR oxygen* [Title/Abstract] OR viability [Title/Abstract] OR phenotype [Title/Abstract] OR differentiation [Title/Abstract] OR matrix [Title/Abstract]) AND (“2016/01/01” [Date—Publication]: “2026/08/09” [Date—Publication]) AND English [Language]
Web of Science Core Collection	TS = (bioprint* OR “extrusion bioprinting” OR “embedded bioprinting” OR “coaxial bioprinting”) AND TS = (bioink* OR “cell-laden hydrogel*” OR “cell laden hydrogel*”) AND TS = (“post-print*” OR maturation OR culture OR crosslink* OR swelling OR degradation OR contraction OR “stress relaxation” OR mechanic* OR interface* OR transport OR perfusion OR oxygen* OR viability OR phenotype OR differentiation OR matrix) AND PY = (2016–2026) AND LA = (English)
Scopus	TITLE-ABS-KEY (bioprint* OR “extrusion bioprinting” OR “embedded bioprinting” OR “coaxial bioprinting”) AND TITLE-ABS-KEY (bioink* OR “cell-laden hydrogel*” OR “cell laden hydrogel*”) AND TITLE-ABS-KEY (“post-print*” OR maturation OR culture OR crosslink* OR swelling OR degradation OR contraction OR “stress relaxation” OR mechanic* OR interface* OR transport OR perfusion OR oxygen* OR viability OR phenotype OR differentiation OR matrix) AND PUBYEAR > 2015 AND PUBYEAR < 2027 AND (LIMIT-TO (LANGUAGE, “English”))
